# Reduction in Hospital Admissions for Cardiovascular Diseases (CVDs) during the Coronavirus Disease 2019 (COVID-19) Pandemic: A Retrospective Study from a Southern Italian Region in the Year 2020

**DOI:** 10.3390/healthcare10050871

**Published:** 2022-05-09

**Authors:** Fabrizio Cedrone, Giuseppe Di Martino, Pamela Di Giovanni, Emilio Greco, Edoardo Trebbi, Ferdinando Romano, Tommaso Staniscia

**Affiliations:** 1 Local Health Authority of Pescara, Via Paolini, 65100 Pescara, Italy; cedronefab@gmail.com; 2 Department of Pharmacy, “G. d’Annunzio” University of Chieti-Pescara, Via dei Vestini 31, 66100 Chieti, Italy; pamela.digiovanni@unich.it; 3 Faculty of Innovative Technologies for Digital Communication, Link Campus University, Via del Casale di San Pio V 44, 00165 Roma, Italy; e.greco@unilink.it; 4 Department of Infectious Diseases and Public Health, “La Sapienza” University of Rome, Piazza Aldo Moro 5, 00138 Rome, Italy; edoardo.trebbi@uniroma1.it (E.T.); ferdinando.romano@uniroma1.it (F.R.); 5 Department of Medicine and Ageing Sciences, “G. d’Annunzio” University of Chieti-Pescara, Via dei Vestini 31, 66100 Chieti, Italy; tommaso.staniscia@unich.it

**Keywords:** COVID-19, cardiovascular disease, hospital admission, healthcare services, HDR, Italy

## Abstract

(1) Background: The COVID-19 pandemic has indirect consequences for healthcare for other diseases, known as collateral damage. This situation heavily affects healthcare systems, causing changes in patterns of hospital admission. During the peak of the coronavirus disease 2019 pandemic, numerous studies reported a reduction in admissions for acute coronary syndrome. The aim of this study was to evaluate the incidence of admissions for cardiovascular diseases in Abruzzo, a region of Southern Italy, in the year 2020 and compare it to the two previous years (2018–2019). (2) Methods: This retrospective study was conducted in Abruzzo, Italy. The monthly number of admissions in the year 2020 was compared to a control period made from the average number of events that occurred in the previous two years (2018–2019). (3) Results: A global reduction in hospital admissions for all the cardiovascular diseases (CVDs) considered was observed. In particular, compared to the control period, in 2020, the number of admissions for ST-segment elevation myocardial infarction (STEMI) was lower by 34 (hospitalization rate ratio, HRR, 0.93; *p* < 0.001), the number of non-ST-segment-elevation myocardial infarctions (N-STEMI) was lower by 154.5 (HRR 0.89; *p* < 0.001), the number for acute coronary syndrome (ACS) was 340 lower (HRR 0.90; *p* < 0.001) and the number for heart failure (HF) was 1424.5 lower than during the control period (HRR 0.73; *p* < 0.001). (4) Conclusions: The results of this study show the impact of COVID-19 on admissions for CVDs, suggesting the need for strategic measures to overcome the burden of hospitalizations in future years.

## 1. Introduction

From December 2019, an emerging infectious disease caused by coronavirus disease 2019 (COVID-19) rapidly spread around the globe [1,2]. It was subsequently classified as a pandemic by the World Health Organization (WHO) [3]. The pandemic heavily affected health systems, with high hospitalizations and fatality rates. In addition to the direct impact of COVID-19 on morbidity and mortality, the pandemic had indirect effects on healthcare for other diseases, known as collateral damage [4]. In Italy, after the infectious disease spread, the government adopted a state-wide lockdown. This measure, along with the conversion of several hospitals in specific centers to treat only COVID-19 patients, led to a drastic reduction in non-COVID-19 specialist outpatient performances. This situation heavily affected the healthcare system, causing changes in patterns of hospital admission [4,5]. Routine diagnostic procedures and elective admissions were cancelled or postponed due to the focus on the care of patients with COVID-19 [6]. During the peak of the COVID-19 pandemic, numerous hospitals across Europe and the United States of America (USA) reported a reduction in admissions for acute coronary syndrome (ACS), which correlates with higher case fatality and complication rates [4,5,7]. These situations cause concern among physicians and public health authorities about the prognosis of patients with ACS and other cardiovascular diseases (CVDs) because the outcomes of these events depend largely on rapid diagnosis and therapy [7]. Previous studies reported a reduction in admissions for CVDs in several hospitals in Europe [8] and Italy [5], but none of these reported data from an entire region through comparison with two previous years from pandemic. The aim of this study was to evaluate the incidence of admissions for CVDs in a Southern Italian Southern region in the year 2020 and compare it to the two previous years (2018–2019).

## 2. Materials and Methods

### 2.1. Study Design

This retrospective study was conducted in Abruzzo, a region of Southern Italy with about 1.3 million inhabitants. The number of CVD admissions (as indicated below) that occurred in the year 2020 was studied and compared to the average number of the same events that occurred in the previous two years (2018–2019) [9,10]. Data were collected from hospital discharge records (HDR), which included information on patients’ demographic characteristics, the diagnosis-related group (DRG) used to classify the admission and a maximum of six diagnoses (one principal diagnosis and up to five comorbidities) and six procedures that occurred during the hospitalization. Diagnoses and procedures were coded according to the International Classification of Disease, 9th Revision, Clinical Modification (ICD-9-CM), the National Center for Health Statistics (NCHS) and the Centers for Medicare and Medicaid Services External, Atlanta, GA, USA. The study considered cardiovascular hospitalizations concerning the following categories and through the following coding:

- Acute coronary syndrome (ACS): discharge with a principal diagnosis of ICD-9-CM 410.xx, 411.xx, or 413.xx
- ST-segment elevation myocardial infarction (STEMI): discharge with a principal or secondary diagnosis of ICD-9-CM 410.xx, excluding those with principal or secondary diagnosis of 410.7x or 410.9x
- Non-ST-segment elevation myocardial infarction (N-STEMI): discharge with principal or secondary diagnosis of ICD-9-CM 410.7x, excluding those with a principal or secondary diagnosis of 410.9x
- Heart failure (HF): discharge with a principal diagnosis of ICD-9-CM 398.91, 402.01, 402.11, 402.91, 404.01, 404.03, 404.11, 404.13, 404.91, 404.93, 428.0, 428.1, 428.2x, 428.3x, 428.4x, or 428.9.

Monthly number of admissions in the year 2020 was compared to the equivalent value for a control period comprising the average number of events that occurred in the previous two years (2018–2019).

### 2.2. Statistical Analysis

Quantitative variables were summarized as mean and standard deviation (SD) or median and interquartile range (IQR), according to their distribution. Qualitative variables were summarized as frequency and percentage. Incidence rate ratios comparing the study period with each of the control periods were expressed as Hospital Rate Ratio (HRR) with a 95% confidence interval (95% CI) and were calculated using the Poisson regression, adjusted for age, gender and hospital. For all analyses, a *p*-value ≤ 0.05 was assumed to be statistically significant (two-tailed). Statistical analyses were performed using STATA v14 software (StataCorp LLC, College Station, TX, USA).

## 3. Results

During the study period in the Abruzzo region, 1019 patients were admitted for STEMI, 1248 patients were admitted for N-STEMI, 3809 patients were admitted for ACS and 3736 patients were admitted for HF, as reported in Table 1.

A global reduction in hospital admissions for all the CVDs considered was observed, as reported in Table 2. In particular, compared to the control period, in 2020, there were 34 fewer admissions for STEMI (HRR 0.93; 95% CI 0.92–0.94; *p* < 0.001), the value for N-STEMI was lower by 154.5 (HRR 0.89; 95% CI 0.88–0.90; *p* < 0.001) and the value for ACS was lower by 340 (HRR 0.90; 95% CI 0.89–0.90; *p* < 0.001). The main reduction was observed for the HF admissions, which were lower by 1424.5 than during the control period (HRR 0.73; 95% CI 0.73–0.73; *p* < 0.001).

**Figure 1:**
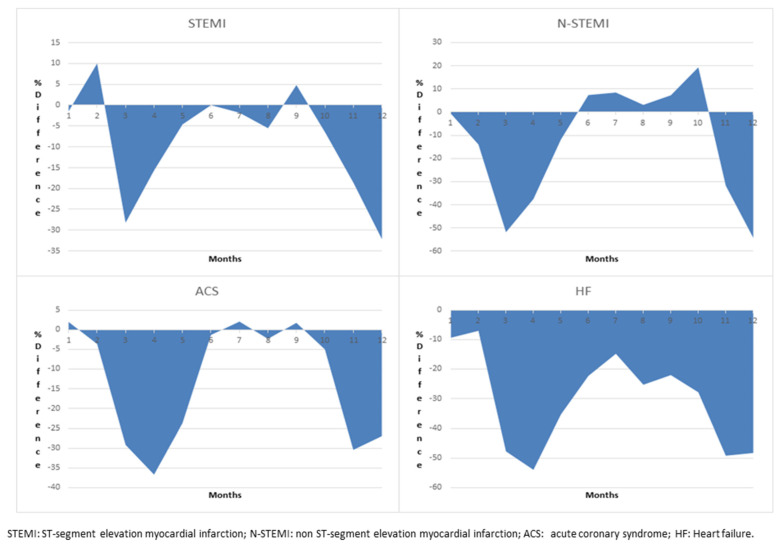
Monthly differences in CVD admissions by month in 2020.

No differences were found in terms of length of stay (LOS) or in-hospital mortality during the study periods (Table 1). At the same time, a significant difference was found in the patients’ age, with a reduction in hospitalization, particularly for patients aged less than 75 years. According to the monthly incidence of CVD admissions, the strong reduction was observed between February and June 2020 and during the last three months of the year, as shown in Figure 1.

## 4. Discussion

The COVID-19 pandemic has placed a heavy burden on hospitals across the world. This study assessed hospital admissions for CVDs using HDR in a region of Southern Italy, in comparison with two previous years. During the COVID-19 pandemic in 2020, there was a significant decrease in hospital admissions for CVDs compared to the previous two years in the Abruzzo region, in Italy. This result is in line with the previous literature, confirming findings from across Italy and Europe [4,5,11]. There was a clear impact at the start of the lockdown, with no patients with CVDs being admitted to hospital during the first period of the year. This can be explained by the presence of a state lockdown in Italy, reflecting the general population’s fear of using emergency services [12]. In order to explain these decreases, it should be considered that patients might be reluctant to seek hospital care for fear of infection or contagion [13,14]. A previous cross-sectional study conducted in the United Kingdom showed that patients were afraid of being exposed to COVID-19 and were worried of about increasing the pressure on the United Kingdom’s already overburdened healthcare system [15,16]. The reluctance to seek emergency care seems to be more prevalent in less severe cases [17]. The observed reduction in admissions could be also explained by the adaptation of the healthcare system to the pandemic. Higher limits for referrals to hospitals or emergency departments, lower intensive care unit (ICU) capacity, declines in ambulatory cardiovascular visits, or delays in the treatment of less urgent cases can all cause an overall reduction in hospital admissions [7,18]. On the other hand, previous studies suggested the presence of an increased severity of COVID-19-related symptoms in patients with CVDs [18]. This is also supported by the findings of a study from China, where COVID-19 patients who required intensive care admissions were more likely to suffer from CVDs than non-ICU patients [19]. As consequence, this could increase the risk of patients admitted for COVID-19 symptoms being undiagnosed due to the medical focus on COVID-19. With regard to gender, several studies investigated the sex differences in the impact of COVID-19 on the care and management of ACS [5,20,21,22,23,24,25,26], observing no difference between genders, whereas two studies reported a reduction in STEMI-related admissions among women compared to men [5,22]. Clinicians raised a significant concern about the increased number of cardiovascular cases in hospitals in the future due the presentations that were previously hidden or stable during the pandemic period. In addition, as a consequence of this crisis, in the immediate future, there could be a surge of patients with delayed complications of ACS and reinfarctions [27]. The impact of COVID-19 also affected other conditions, such as strokes [28] and kidney disease [29]. In addition, the social distancing policy caused a lower number of admissions for injuries and traffic accidents [30].

The strength of this study is the large sample analyzed and the long study period considered. This is the first study to have been conducted in Italy considering all the hospital admissions occurring in an entire region. In addition, this study compared the incidence in 2020 with those of two different control years. In comparison with previous studies conducted in Italy and the UK [26], this study confirmed the reduction in both STEMI and non-STEMI admissions. In addition, this is one of first studies to also investigate HF admissions.

However, the results of this study should be considered in the light of the following limitations. Firstly, the selection of the diagnoses was based on ICD-9-CM codes that did not consider the severity of the diseases. Second, the use of administrative data may have been limited by the lack of some clinical information, while some diagnostic codes could have been under-reported or incorrect. Third, information about out-of-hospital mortality was lacking, so the excess mortality during the study period cannot be calculated.

## 5. Conclusions

The results of this study have immediate relevance for cardiovascular clinicians and public health authorities. They showed the impact of the COVID-19 pandemic on admissions for CVDs, highlighting the need for strategic measures to overcome the burden of hospitalizations future years, especially in the event of future lockdowns, in order to raise the awareness of health professionals and educate patients to call the emergency services if cardiac symptoms occur.

## Figures and Tables

**Table 1:** Comparison of admissions that occurred between 2020 and the mean of the years 2018–2019.

	2018	2019	Mean between 2018–2019 (SD)	2020	Diff.	*p*-Value *
STEMI						
Admissions	1096	1010	1053 (11.7)	1019	−34.0	
Age						
18–44	35	168	101.5 (8.7)	37	−64.5	<0.001
45–74	669	492	580.5 (8.9)	653	72.5	
≥75	392	350	371 (16.7)	329	−42.0	
LOS median (IQR)	6.5 (5–10)	6 (5–9)	6 (5–10)	6 (5–9)		0.146
Death (N%)	115	112	113.5(10.8)	107(10.5)	−6.5	0.808
**N-STEMI**						
Admissions	1.512	1.293	1402.5 (20.8)	1248	−154.5	
Age						
18–44	38	217	127.5 (18.2)	19	−108.5	<0.001
45–74	776	627	701.5 (18.3)	687	−14.5	
≥75	698	449	573.5 (26.1)	542	−31.5	
LOS median (IQR)	6 (4–10)	6 (4–9)	4 (2–10)	6 (4–9)		0.444
Death (N%)	79	80	79.5 (5.7)	62 (5)	−17.5	0.546
**ACS**						
Admissions	4489	3809	4149 (26.2)	3809	−340.0	
Age						
18–44	103	622	362.5 (25.3)	77	−285.5	<0.001
45–74	20,794	1929	2361.5 (25.6)	2407	45.5	
≥75	1592	1258	1425 (61.2)	1325	−100.0	
LOS median (IQR)	5 (2–8)	5 (2–8)	5 (2–8)	5 (2–8)		0.009
Death (N%)	200	199	199.5 (4.8)	161 (4.2)	−38.5	0.382
**HF**						
Admissions	5738	4583	5160.5 (78.6)	3736	−1424.5	
Age						
18–44	31	763	397 (69.8)	28	−369.0	<0.001
45–74	1.296	1.925	1610.5 (68.5)	1047	−563.5	
≥75	4.411	1.895	3153 (96.9)	2661	−492.0	
LOS median (IQR)	8 (5–12)	8 (5–12)	8 (5–12)	8 (5–12)		0.615
Death (N%)	536	426	481 (9.32)	321 (8.59)	−160.0	0.482

* Absolute difference between year 2020 and the mean of years 2018–2019. STEMI: ST-segment-elevation myocardial infarction; N-STEMI: non-ST-segment-elevation myocardial infarction; ACS: acute coronary syndrome; HF: Heart failure; LOS: length of stay; IQR: interquartile range.

**Table 2:** Hospitalization rate ratios of CVDs in 2020 compared to the two-year period of 2018–2019.

	HRR (95% CI)	*p*-Value * ^+^
	STEMI	
2020 vs. 2018–2019	0.93 (0.92–0.94)	<0.001
Male vs. female	0.99 (0.98–1.00)	0.331
Age		
18–44	Ref.	
45–74	0.99 (0.98–1.00)	0.523
≥75	0.99 (0.98–1.01)	0.948
**N-STEMI**		
2020 vs. 2018–2019	0.89 (0.88–0.90)	<0.001
Male vs. female	1.00 (0.99–1.00)	0.338
Age		
18–44	Ref.	
45–74	0.99 (0.97–1.00)	0.062
≥75	0.98 (0.97–0.99)	0.015
**ACS**		
2020 vs. 2018–2019	0.90 (0.89–0.90)	<0.001
Male vs. female	1.00 (1.00–1.00)	0.003
Age		
18–44	Ref.	
45–74	0.99 (0.99–1.00)	0.925
≥75	1.00 (0.89–0.90)	0.978
**HF**		
2020 vs. 2018–2019	0.73 (0.73–0.73)	<0.001
Male vs. female	1.00 (0.99–1.00)	0.073
Age		
18–44	Ref.	
45–74	1.00 (0.99–1.00)	0.312
≥75	1.00 (0.99–1.00)	0.186

* All models were adjusted for hospital. ^+^ Poisson regression. STEMI: ST-segment-elevation myocardial infarction; N-STEMI: non-ST-segment-elevation myocardial infarction; ACS: acute coronary syndrome; HF: Heart failure; LOS: length of stay; 95% CI: 95% confidence interval; IQR: interquartile range.

## Data Availability

Data available on request due to privacy and ethical restrictions.
